# The Effect of Oral Gabapentin and Pregabalin as a Prodrug in Pain Control after Orthopedic Surgery on the Upper Limb: A Double-Blind Parallel Randomized Clinical Trial Study

**DOI:** 10.1155/2024/7193599

**Published:** 2024-05-06

**Authors:** Lida Nouri, Rana Roshanfekr, Azam Biderafsh, Reza Pakzad, Hamed Azadi

**Affiliations:** ^1^Department of Anesthesiology, Medicine Faculty, Ilam University of Medical Sciences, Ilam, Iran; ^2^Zoonotic Disease Research Center, Ilam University of Medical Sciences, Ilam, Iran; ^3^Student Research Committee, Ilam University of Medical Sciences, Ilam, Iran; ^4^Department of Epidemiology, School of Public Health, Tehran University of Medical Sciences, Tehran, Iran; ^5^Department of Epidemiology, Faculty of Health, Ilam University of Medical Sciences, Ilam, Iran; ^6^Department of Anesthesiology, School of Allied Medical Sciences, Ilam University of Medical Sciences, Ilam, Iran

## Abstract

**Objective:**

To compare the effects of oral gabapentin (GBP) and pregabalin (PGB) in pain control after orthopedic surgery on the upper limb.

**Methods:**

In this double-blind randomized clinical trial study, 80 patients who were the candidates for elective orthopedic surgery on one of the parts of the upper limb were divided into two groups using balance-block randomization. For the first group, a 150 mg capsule of PGB (one hour before the surgery) and for the second group, a 300 mg capsule of GBP (two hours before the surgery) were prescribed. Patients were subjected to standard monitoring at the beginning and during surgery. The pain scores were evaluated at before surgery, in PACU (postanesthesia care unit), and 6 and 12 hours after the surgery by VAS (visual analog scale).

**Results:**

In this study, 37 subjects were allocated to each group. The participation rate was 92.5%. The mean with 95% confidence interval (CI) of pain scores over 4 times in the PGB group was 4.03 (3.25–4.79), 3.76 (3.02–4.49), 3.65 (3.06–4.23), and 3.41 (2.88–3.93) and in the GBP group was 4.08 (3.33–4.83), 2.78 (2.11–4.45), 2.3 (2.05–2.54), and 2 (1.51–2.50), respectively. The within-group comparisons showed a significant decrease in the pain score over time (*P* < 0.001). Also, the between-group comparison showed significant differences between the two groups in terms of pain score (*P* < 0.001). In the end, results showed that there is a significant interaction between time and intervention for pain score (*P*=0.042).

**Conclusion:**

Although two medicines led to a reduction in the pain score, but the rate reduction in the PGB group was significantly more than that in the GBP group. This trial is registered with IRCT20211013052759N1.

## 1. Introduction

Acute pains after surgery lead to physical complications, increase in metabolism and blood pressure, and exacerbation of underlying diseases, which ultimately increases the length of hospital stay, increases patient costs, patient dissatisfaction, and causes chronic pains [1, 2].

There are various methods to control pain following surgery, which mainly use nonsteroidal anti-inflammatory drugs (NSAIDs). Also, oral pain relievers such as acetaminophen in combination with different opioid derivatives can reduce pain with different mechanisms [3]. Even though very effective in treating pain, possible side effects, including respiratory depression, nausea, and vomiting, limit opioid use in postoperative pain management [4, 5]. Many studies have investigated the use of other drugs to reduce narcotic consumption for pain control [6].

Among the drugs used for pain control are pregabalin (PGB) and gabapentin (GBP) of the gabapentinoid family [7, 8]. GBP is an alkylated analog of the neurotransmitter gamma-aminobutyric acid (GABA), which is used in the treatment of neuropathic pain, epilepsy, and anxiety [9–11]. PGB is another analog of GABA [12]. By binding presynaptically to a part of the voltage-dependent calcium channel, both drugs cause less release of excitatory neurotransmitters such as glutamate [13], norepinephrine (NE), substance P (SP), and calcitonin gene-related peptide (CGRP), and ultimately reduce pain and its central perception [14, 15].

In addition to their analgesic effects, nonopioid analgesics, such as PGB and GBP, can reduce the need for opioids, lower the opioid dosage, and minimize the side effects associated with opioid use [16, 17]. Gabapentinoids are generally well tolerated, however, PGB causes slight dizziness and drowsiness, but has no effect on blood pressure and heart rate [13]. In addition to drowsiness and dizziness, GBP can cause peripheral edema [18–20]. According to the available data, GBP and PGB can reduce the possibility of delirium and the amount of vomiting after surgery [21], which is usually caused by opioids [22]. Although PGB and GBP are very similar in terms of antiepileptic, analgesic, and antianxiety properties, PGB has better pharmacokinetics including dose-independent absorption [23, 24] and usually with a much lower dose, it has the same effect as GBP and side effects are less [12].

Various studies have shown that drugs such as gabapentin (GBP) and pregabalin (PGB) can be effective in reducing the severity of acute postoperative pain and reducing the need for opioids. They may also play a role in preventing chronic postoperative pain [11, 25–27]. A systematic review study found that the amount of pain reduction was greater in the group receiving GBP in four studies and PGB in three studies, compared to the comparison group [26]. Another study found that the duration of analgesia (pain relief) in the PGB group was twice as long as the GBP group [25]. The results of another study in Iran also showed that the rate of pain reduction in the PGB group was higher than that of the GBP group [11].

These findings suggest that GBP and PGB may be effective and safe drugs for reducing postoperative pain. However, more research is needed to confirm these findings. Several studies have shown that GBP and PGB, which are prodrugs, can reduce pain after surgery and the need for narcotics [1, 11, 25–28]. These drugs have also been shown to have positive effects on patients' hemodynamic changes during laryngoscopy and other surgeries [1]. Although the results of these studies show the beneficial effects of PGB and GBP [27, 28], they involved lower limb surgeries and laparoscopy [10]. In past studies, these two drugs have not been compared with each other in orthopedic surgery on the upper limbs. For this purpose, we established the hypothesis that oral gabapentin (GBP) and pregabalin (PGB) affect pain reduction after orthopedic surgery on the upper limb. Therefore, the present study aimed to fill that gap by comparing the independent and combined effects of GBP and PGB on reducing postoperative pain following upper limb surgery.

## 2. Methods

### 2.1. Setting

This was a double-blind randomized clinical trial implemented in Imam Khomeini Hospital in Ilam city in 2022 through a two-arm parallel design.

### 2.2. Sample Size

According to the following formula, and considering that the mean pain score in the methadone group was 6.25 ± 3.09 and the diclofenac group was 4.57 ± 2.16 [29], *β* = 20%, and *α* = 5%, the sample size was estimated to be 40 in each group (80 individuals in total).

(1) $$\begin{array}{c} n=\frac{{\left(Z_{1-\alpha /2}+Z_{1-\beta }\right)}^{2}*\;\left(S_{1}^{2}+S_{2}^{2}\right)}{{\left(\mu_{1}-\;\mu_{2}\right)}^{2}}\\ =\frac{\;{\left(1.96+0.84\right)}^{2}*\;\left(3.09^{2}+2.16^{2}\right)}{{\left(6.25-\;4.57\right)}^{2}}=40. \end{array}$$

### 2.3. Inclusion and Exclusion Criteria

Inclusion criteria were as follows: age range of 20–55 yrs, candidate of elective orthopedic surgery on one of the parts of the upper limb with general anesthesia, and ASA class I or II (ASA I: healthy patient without organic disease and ASA II: patient with mild systemic diseases whose disease does not affect his daily activities). The exclusion criteria were as follows: unwillingness to study, severe hemodynamic instability, neuropsychological diseases, history of seizures, acute or chronic kidney diseases, alcohol or drug use, and history of sensitivity to GBP or PGB.

### 2.4. Sampling and Random Assignment

**Figure 1:**
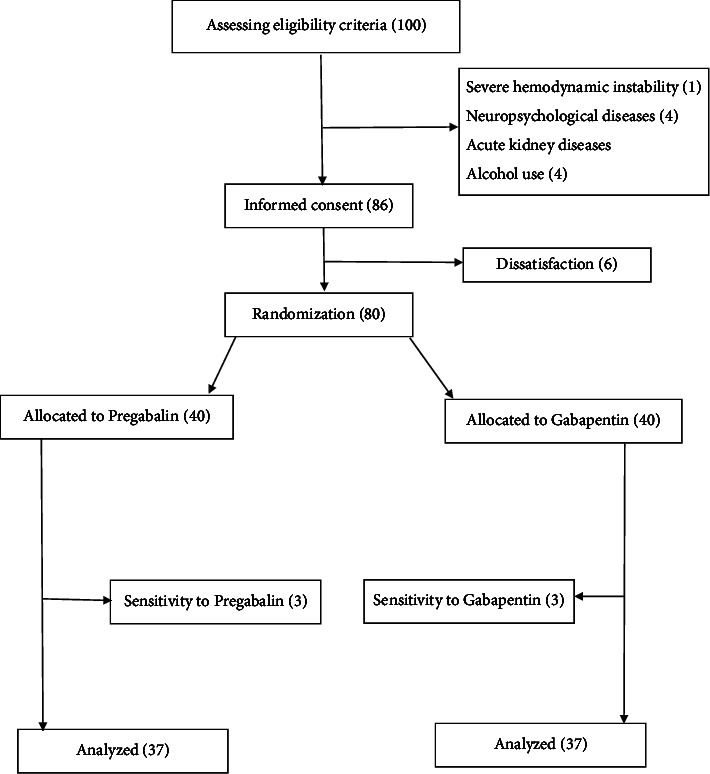
Flowchart of the study design.

Initially, 100 patients who were admitted to the hospital for elective orthopedic surgery on one of the parts of the upper limb were selected. After applying the study's inclusion and exclusion criteria, 86 patients remained. These patients were given a full explanation of the study's goals and were asked to provide informed consent. Six patients refused to consent after hearing the explanation and were excluded from the study (Figure 1). Finally, 80 patients were assigned to the PGB and GBP groups using balance-block randomization in blocks of 4. The “ralloc” package in Stata software was used to create the random blocks. The participant's enrollment was performed by L-N and R-R and the random allocation sequence was generated by the methodologist coauthor (R-P and A-B).

### 2.5. Concealment and Blinding

Balance-block randomization was applied to ensure that both the participants and the researchers were blind to the treatment allocation. Hence, for concealment, based on a random allocation sequence, a series of encoded randomization envelopes were created so that each code indicated a type of medication. The principle investigator (R-P) opened the recruitment envelopes sequentially and the participant's assignment was determined based on a list of codes that were prepared for each medication. It should be noted that the final code list was at the disposal of the principal investigator and other coauthors did not know about the meaning of each code. Since the assignment of patients was concealed until statistical analysis completion, the participants, anesthesiologist, or person who had the duty to prescribe the medication, and examiner were blinded to the group allocation throughout the trial.

### 2.6. Intervention and Procedure

The baseline pain score was assessed for all patients. A trained Bachelor of Science in anesthesia (R-R), who was blinded to the type of medicine, administered 150 mg of PGB to the first group one hour before surgery and 300 mg of GBP to the second group two hours before surgery. All patients were subjected to standard monitoring at the beginning of entering the operating room, including blood pressure, heart rate, pulse oximetry, and cardiography examination. An intravenous catheter (no.18) was inserted into all patients and 500 ml of Ringer's serum was administered. Midazolam 1 mg was injected as a premedication for all patients. Propofol 2 mg/kg and atracurium 0.5 mg/kg were used to induce anesthesia. Propofol 100 mg/kg/min was used to maintain anesthesia and intermittent amounts of atracurium were used. After rechecking the vital signs and ensuring the appropriate depth of anesthesia, surgeons were given permission to begin the surgery.

It should be noted that surgery was performed by several orthopedic surgeons under a unique approach. After the end of the surgery, the patients were transferred to the recovery room, and then, the patients were evaluated at three times including before surgery, in PACU (postanesthesia care unit), and 6 and 12 hours after the surgery by the nurse. During the evaluation, the pain score was assessed by VAS (visual analog scale). If the patients had vomiting or nausea at PACU, the antiemetic drugs were prescribed. As well as, if the patients had a pain score of 3 or more, they received the same dose of intravenous morphine, but for the first time they were needed to receive morphine, and the number of times they received morphine after the operation and the duration the patients stayed in recovery were recorded.

### 2.7. Statistical Analysis

Data were analyzed using Stata version 11 by considering the intention-to-treat (ITT) approach. The Kolmogorov–Smirnov test assessed the normality of the data distribution. The Student's *t*-test and chi-square test compared the baseline data. One-way repeated measure ANOVA was used to compare the effect of the interventions at the three time points, and the Bonferroni test was used for pairwise comparisons. Data are presented as mean ± SD for quantitative variables and number (%) for qualitative variables. The significance level was set at *P* < 0.05.

## 3. Results

### 3.1. Comparing Baseline Data on the Two Groups

Given that after the random allocation of subjects in the study groups, 3 patients in each group experienced sensitivity to PGB/GBP. Finally, 37 patients were compared in each group. The participation rate was 92.5%. All the information was measured and there was no information missing in this regard. All quantitative variables were normal. The mean age with 95% confidence interval (CI) of the subjects in GBP and PGB were 41.32 (36.91–45.74) and 41.08 (36.49–45.67), respectively. The mean BMI in GBP and PGB were 25.79 (24.27 to 27.31) and 25.98 (24.77 to 27.18), respectively. 48.64% (32.04–65.25) patients in GBP and 51.35% (34.75–67.95) patients in PGB groups were male. Comparing baseline variables between the two groups of GBP and PGB showed no significant difference in variable age (*P*=0.686), BMI (*P*=0.952), and gender (*P*=0.538) between the two groups. The white blood cell (WBC; *P*=0.152), red blood cell (RBC; *P*=0.850), and hemoglobin (Hb; *P*=0.542) levels were not significantly different between the two groups. Prothrombin time (PT: *P*=0.456), partial thromboplastin time (PTT; *P*=0.483), and international normalized ratio (INR; *P*=0.289) were not significantly different between the two groups. The means of the other variables are shown in Table 1.

### 3.2. Intraparticipant Variability (Time Effect) in the Pain Scores

**Figure 2:**
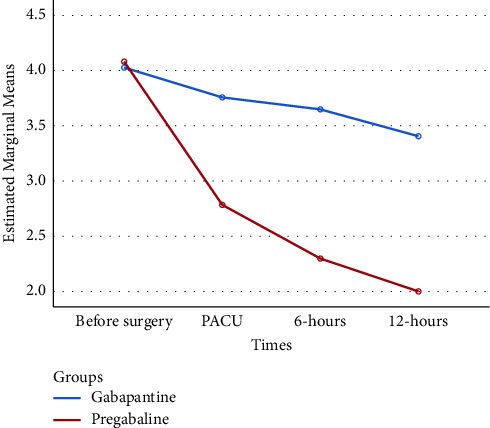
Average of the pain score variable between the gabapentin and pregabalin groups at four time points measured.

Comparing the average variable pain score in four time measurements showed that the average variable pain score in the GBP group decreased over time so it was 4.03 (95% CI: 3.25–4.79) at the first time, 3.76 (95% CI: 3.02–4.49) at the second time, 3.65 (95% CI: 3.06–4.23) at the third time, and 3.41 (95% CI: 2.88–3.93) in fourth time. These values for the PGB group were 4.08 (95% CI: 3.33–4.83), 2.78 (95% CI: 2.11–4.45), 2.3 (95% CI: 2.05–2.54), and 2 (95% CI: 1.5–2.50), respectively. This pattern is shown in Table 2 and Figure 2. Results of within-subject in repeated measure ANOVA showed a significantly different pain score over time (*P* < 0.001). Bonferroni test results for pairwise comparison showed that pain scores at the third time (*P*=0.003) and fourth time (*P* < 0.001) were less than the first time. The other pair comparison was not statistically significant (Table 3).

### 3.3. Intraparticipant Intervention Effect on the Pain Scores

Overall, the average score of pain in the GBP and PGB groups were 3.71 (95% CI: 3.06–4.36) and 2.79 (95% CI: 2.25–3.33), respectively. The result of between subjects in repeated measure ANOVA showed that the pain score in PGB was statistically significantly lower than GBP (*P* < 0.001) (Table 2 and Figure 2).

### 3.4. The Interaction between the Intervention Time and Pain Score

The results showed a significant association between the intervention time and pain score (*P*=0.042). In particular, the pain score reduction rate in the PGB group was significantly higher than that in the GBP group (Table 2).

### 3.5. Comparison of Liver and Renal Functions and Other Side Effects between the Two Groups

Tale 4 shows the incidence of side effects in the two study groups. As shown, there are no differences between the two groups corresponding to dizziness (*P*=0.772), drowsiness (*P*=0.553), nausea (*P*=0.639), and vomiting (*P*=0.496). Other side effects are shown in Table 4. Also based on Table 4, the intervention did not have considerable effects on liver and renal functions so the values of liver function and renal function indices were in normal ranges and also there were no differences between the two groups.

## 4. Discussion

Surgical patients usually experience severe pain within the first 24 hours after surgery. Thus, using effective drugs with few side effects for postoperative pain management is very important. In this study, the assessment of pain score was performed in 1st, 2nd, 3rd, and 4th time postoperatively. The within-group comparison showed a significant decrease in the pain score over time (*P* < 0.001). Also, between-group comparisons showed significant differences between the two groups in terms of pain score (*P* < 0.001). The pain score was significantly reduced in the pregabalin group compared to the gabapentin group (*P* < 0.001). The change in pain intensity and the amount of pain reduction over time was significant in both groups. Specifically, the pain score in the GBP group reduced from 4.03 to 3.41, and in the PGB group, it reduced from 4.08 to 2.0. Our results are consistent with those of Davari et al. [30], Robertson et al. [31], Tiippana et al. [27], Mahoori et al. [1], and other similar studies [11, 25–27]. The analgesic mechanism of PGB and GBP is that by presynaptic connection to a part of the voltage-dependent calcium channel, causes less release of excitatory neurotransmitters and ultimately reduces pain and central perception [7, 10].

The results of our study showed that PGB was more effective at reducing pain than GBP. The average pain score in the GBP group was 3.71, while the average pain score in the PGB group was 2.79. The results of repeated measure ANOVA showed that the interaction term between time and drug was significant, which means that the speed of pain reduction was faster in the PGB group than in the GBP group. The pain score in the PGB group decreased from 4.08 to 2, while the pain score in the GBP group decreased from 4.03 to 3.41. This finding is consistent with other studies [32, 33]. For example, a study by Kheirabadi et al. [32], found that in the orthopedic surgery of the lower limb, the intensity of pain in the group receiving PGB at the time of recovery compared to the control group was significantly less, but other drugs (GBP and celecoxib) did not significantly reduce pain during the recovery period. The PGB group required significantly less pethidine dose during admission to the surgical ward, while the placebo group required the most.

Also, Saraswat and Arora [33] showed that the duration of analgesia for acute pain after surgery under spinal anesthesia was 8.98 hours in the GBP 1200 mg group and 14.17 hours in the PGB 300 mg group, which showed that PGB is more effective. Hasani et al. [11] showed that the observed difference between GBP and PGB in recovery time was statistically significant. The lowest pain intensity was in the PGB group and the lowest observed difference was with the dose of 900 mg. Nevertheless, it was shown in Mahoori et al.'s study [1] that the effect of GBP in suppressing hemodynamic responses was more prominent than that of PGB. Although the heart rate and systolic and diastolic blood pressure during the study period were lower than the control group, but this difference was not significant and the cause of this problem could be the inability to equate the dose of the drugs.

The main difference in the effectiveness of GBP and PGB is more related to the bioavailability of these two drugs than to their mechanism of action. PGB with a bioavailability of 90% is quickly absorbed orally and reaches its maximum plasma level within 30 minutes to 2 hours [34].

GBP and PGB are both drugs that are absorbed in the small intestine. However, GBP is only absorbed in a limited part of the duodenum, while PGB can be absorbed throughout the entire small intestine. This means that GBP has a maximum absorption capacity, while PGB does not [35, 36]. When the absorption capacity of GBP is saturated, subsequent doses of the drug will cause a gradual and slow increase in the blood concentration of GBP. However, each dose increase of PGB will be accompanied by an increase in blood concentration, as PGB can be absorbed throughout the entire small intestine [37]. This difference in absorption rates has implications for the therapeutic effects and side effects of GBP and PGB. GBP is expected to have a limit to its therapeutic effects and side effects, as the blood concentration of the drug cannot increase indefinitely. However, PGB does not have this limit, and its therapeutic effects and side effects can increase with each dose increase [22, 38].

It should be noted that there was no contamination or exchange in the study arms after randomization. Therefore, there was no need to conduct a per-protocol analysis. In other words, we had to exclude 3 participants in each group after randomization because they were sensitive to interventions. RCT study and randomization process are the largest strengths of this study. Additional methodological elements such as allocation concealment, blinding, measuring compliance, controlling for cointerventions, and analyzing results by intention-to-treat approach were other strengths of this study. This study, like other studies, has limitations that should be taken into account in the interpretation and use of the findings. This study had some limitations. We did not examine different doses of the drugs. Therefore, the complications caused by higher doses could not be identified. The shorter duration (the first 12 h after surgery) of drug effectiveness evaluation also poses an issue. As a methodological view, the results of this study cannot be generalized to other surgeries.

## 5. Conclusion

The results of this study demonstrated that oral PGB is more effective than GBP in reducing postoperative pain following orthopedic surgery of the upper limb. Questions remain about the effects of different doses of PGB and GBP on postsurgical pain; hence, it warrants further investigations. Furthermore, considering the importance of pain control after surgery, studies that examine postoperative pain after different surgeries and the effect of different doses of drugs over time concerning both the analgesic effect and adverse side effects at higher doses are necessary [39].

## Figures and Tables

**Table 1:** Comparison of basic variables between the two groups^a^.

Variables		Gabapentin (*N* = 37)	Pregabalin (*N* = 37)	*P* value
Background variables	Age (yrs. old)	41.32 (36.91–45.74)	41.08 (36.49–45.67)	0.686
	BMI (kg/m^2^)	25.79 (24.27–27.31)	25.98 (24.77–27.18)	0.952
	Height (m)	168.22 (164.83–171.61)	167.35 (164.04–170.66)	0.970
	Weight (kg)	86.81 (59.34–114.28)	72.35 (67.74–76.96)	0.309
	Male gender	48.64% (32.04–65.25)	51.35% (34.75–67.95)	0.538
	Marital status (married)	75.67% (61.42–89.93)	77.78% (63.77–91.79)	0.338

Hematological variables	WBC (10^3^/*μ*L)	8.51 (7.12–9.9)	10.49 (9.38–11.6)	0.152
	RBC (10^6^/*μ*L)	4.9 (4.51–5.29)	4.87 (4.6 – 5.14)	0.850
	Hb (g/dl)	13.22 (12–14.44)	12.91 (11.93–13.9)	0.542
	HCT (%)	39.85 (37.05–42.64)	38.79 (36.44–41.13)	0.589
	MCV (30)	81.67 (78.02–85.32)	80.06 (76.42–83.69)	0.969
	MCH (pg)	27.01 (25.52–28.5)	25.99 (24.35–27.62)	0.718
	MCHC (g/dl)	33.06 (32.3–33.82)	32.99 (32.27–33.71)	0.629
	PLT	248.91 (180.51–317.31)	250.8 (215.32–286.28)	0.915
	Neutrophil (%)	61.45 (53.69–69.22)	73.27 (68.05–78.49)	0.141
	Lymphocyte (%)	35.55 (27.03–44.06)	26.53 (21.62–31.44)	0.162
	PT (second)	13.31 (12.57–14.04)	13.01 (12.63–13.39)	0.456
	PTT (second)	34.87 (32.06–37.69)	33.67 (31.5–35.84)	0.483
	INR	1.07 (0.99–1.15)	1.03 (1–1.07)	0.289

^a^:Quantitative and qualitative variables were presented as mean/percent with a 95% confidence interval, respectively. Quantitative and qualitative variables were compared between the two groups by using an independent *t*-test and chi-square test, respectively. The significance level was considered as 0.05. BMI, body mass index; WBC, white blood cell; RBC, red blood cell; Hb, hemoglobin; HCT, hematocrit; MCV, mean corpuscular volume; MCH, mean corpuscular hemoglobin; MCHC, mean corpuscular hemoglobin concentration; PT, prothrombin time test; PTT, partial thromboplastin time; INR, international normalized ratio; PLT: platelet count.

**Table 2:** Result of repeated measure ANOVA for comparing the pain score between the two study groups over time.

Times	Gabapentin	Pregabalin	Within-subject effect	Between-subject effect	Interaction
Before surgery	4.03 (3.25–4.79)	4.08 (3.33–4.83)	*F* = 8.28 df = 3, 216 *P* < 0.001^*∗*^	*F* = 615.87 df = 1, 72 *P* < 0.001^*∗*^	*F* = 2.78 df = 3, 216 *P*=0.042^*∗*^
PACU	3.76 (3.02–4.49)	2.78 (2.11–4.45)			
6 hours	3.65 (3.06–4.23)	2.3 (2.05–2.54)			
12 hours	3.41 (2.88–3.93)	2 (1.51–2.50)			
Total	3.71 (3.06–4.36)	2.79 (2.25–3.33)			

The mean (95% confidence interval) studied variables are presented in four time points. ^*∗*^Significance level was considered as 0.05.

**Table 3:** Result of Bonferroni test for pairwise comparison of pain score over time.

Times		*P* value
Before surgery	=PACU	0.201
	>6 hours	0.003^*∗*^
	>12 hours	<0.001^*∗*^

PACU	=6 hours	0.999
	=12 hours	0.165

6 hours	=12 hours	0.874

^*∗*^Significance level was considered as 0.05.

**Table 4:** Liver function, renal function, and other side effects in the two study groups.

Subgroup	Variables	Median ± IQR		*P* value
		Gabapentin (*N* = 37)	Pregabalin (*N* = 37)	
Liver function	AST (U/L)	40 ± 34.5	44 ± 34	0.304^*∗*^
	ALT (U/L)	36 ± 54.5	39 ± 68.5	0.485^*∗*^
Renal function	Cr (mg/dL)	1.3 ± 0.7	1.2 ± 0.65	0.475^*∗*^
	BUN (mg/dL)	17 ± 34.5	18 ± 41	0.623^*∗*^
Other outcomes	NPO (hours)	4.25 ± 0.75	4.25 ± 0.5	0.445^*∗*^

		*N* (%)		
		

Side effects	SBP increase	2 (5.4)	3 (8.1)	0.999^#^
	DBP increase	2 (5.4)	3 (8.1)	0.999^#^
	HR increase	3 (8.1)	2 (5.4)	0.999^#^
	Peripheral edema	0 (0)	0 (0)	—
	Dizziness	8 (21.6)	7 (18.9)	0.772^¶^
	Drowsiness	31 (83.8)	29 (78.4)	0.553^¶^
	Nausea	15 (40.5)	17 (45.9)	0.639^¶^
	Vomiting	4 (10.8)	6 (16.2)	0.496^¶^

^*∗*^Calculated by Mann–Whitney *U* test. ^#^Calculated by Fisher's exact text. ^¶^Calculated by Fisher's exact text. NPO, nil per os (nothing by mouth); SBP, systolic blood pressure; DBP, diastolic blood pressure.

## Data Availability

The .dta data (stata version) used to support the findings of this study are available from the corresponding author upon request.
